# Intratumor Heterogeneity in Uveal Melanoma BAP-1 Expression

**DOI:** 10.3390/cancers13051143

**Published:** 2021-03-07

**Authors:** Gustav Stålhammar, Hans E. Grossniklaus

**Affiliations:** 1 Ocular Oncology and Pathology Service, St. Erik Eye Hospital, 17164 Stockholm, Sweden; 2 Department of Clinical Neuroscience, Karolinska Institutet, 17176 Stockholm, Sweden; 3 Departments of Ophthalmology and Pathology, Emory University School of Medicine, Atlanta, GA 30322, USA; ophtheg@emory.edu

**Keywords:** uveal melanoma, tumor heterogeneity, BAP-1, survival

## Abstract

**Simple Summary:**

Uveal melanoma is the most common primary eye malignancy. In this paper, we examine how the expression of the tumor suppressor BRCA1 associated protein 1 (BAP-1) varies across different parts of these tumors. We find that there are considerable differences, but that these do not matter as long as proper rules for interpretation are applied. Further, we show that analysis of BAP-1 expression adds significant prognostic information to both tumor size and gene expression classifications.

**Abstract:**

Malignant tumors are rarely homogenous on the morphological, genome, transcriptome or proteome level. In this study, we investigate the intratumor heterogeneity of BAP-1 expression in uveal melanoma with digital image analysis of 40 tumors. The proportion of BAP-1 positive cells was measured in full tumor sections, hot spots, cold spots and in scleral margins. The mean difference between hot spots and cold spots was 41 percentage points (pp, SD 29). Tumors with gene expression class 1 (associated with low metastatic risk) and 2 (high metastatic risk) had similar intratumor heterogeneity. Similarly, the level of intratumor heterogeneity was comparable in tumors from patients that later developed metastases as in patients that did not. BAP-1 measured in any tumor region added significant prognostic information to both American Joint Committee on Cancer (AJCC) tumor size category (*p* ≤ 0.001) and gene expression class (*p* ≤ 0.04). We conclude that there is substantial intratumor heterogeneity in uveal melanoma BAP-1 expression. However, it is of limited prognostic importance. Regardless of region, analysis of BAP-1 expression adds significant prognostic information beyond tumor size and gene expression class.

## 1. Introduction

Uveal melanoma is the most common primary intraocular malignancy [1]. Two to four percent of patients have detectable metastases at the time of diagnosis [2]. At a later stage however, more than one third of patients develop unvaryingly fatal metastatic disease [3,4].

Several methods for early prognostication have implemented in clinical practice: Gene expression classification and evaluation of mutations in *BRCA associated protein 1* (*BAP-1*), a tumor suppressor located on chromosome 3p, are two of the strongest prognostic predictors [5,6,7,8,9].

*BAP-1* is one of several genes that undergo mutations to promote epithelial to mesenchymal transition of the tumor cells. In the sequence of mutations, the *BAP-1* mutation has been assumed to occur relatively late, preceded by smaller genomic alterations in G-protein subunits including *GNA11* or *GNAQ* that are present in virtually all uveal melanomas [5]. It is thought that these G-protein mutations are not sufficient for progression to metastatic disease. In contrast, *BAP-1* mutations are thought to appear after the *GNA11* or *GNAQ* mutations, after which the risk for metastatic seeding increases greatly.

Immunohistochemical staining of the BAP-1 protein in tumor tissue and assessment of its level of nuclear expression is a relatively quick and inexpensive alternative to genetic testing, with similar prognostic value [10,11,12]. If digital image analysis or deep learning algorithms are utilized for this assessment, reproducibility is increased [7,8].

Previous studies have shown that there is substantial intratumor heterogeneity in both uveal melanoma phenotype and genotype [13,14,15,16]. If only a smaller portion of a tumor is sampled for prognostic testing including assessment of level of BAP-1 expression, the presence of such heterogeneity may impact the results significantly [12,17,18,19]. In this paper, we therefore investigate how the proportion of BAP-1 positive cells varies across the cut surface of uveal melanomas, and the prognostic weight of different tumor regions.

## 2. Materials and Methods

### 2.1. Patients and Samples

A total of 40 enucleated eyes were included, based on a histologically confirmed diagnosis of malignant melanoma arising from the choroid or ciliary body, availability of gene expression classification and follow-up data. Another 22 eyes had been considered but excluded due to tissue necrosis, bleeding, heavy pigmentation, uneven staining, artefacts or tissue folds in any portion of the tumor section at a degree suspected to interfere with BAP-1 assessment (*n* = 17), or plaque brachytherapy or transpupillary thermotherapy (TTT) prior to enucleation (*n* = 5). Normally, a tissue fold or uneven staining in one minor portion of a tumor does not prevent analysis of the remaining tumor. The relatively high proportion of excluded cases in this project was necessitated by a need for optimal staining of the full tumor section, to enable comparative analysis with hot spots, cold spots and scleral margins.

### 2.2. Immunohistochemistry

The FFPE were cut into 4 μ sections, pretreated in EDTA-buffer at pH 9.0 for 20 min and incubated with mouse monoclonal antibodies against BAP-1 (clone C-4, Santa Cruz Biotechnology, Dallas, TX, USA) at a dilution of 1:40. The dilution had been gradually titrated until optimal staining was achieved, according to manual control by a board certified pathologist (GS). A red chromogen was used. Finally, the sections were counterstained with hematoxylin and rinsed with deionized water. The deparaffinization, pretreatment and staining steps were run in a Bond III automated IHC/ISH stainer (Leica, Wetzlar, Germany).

### 2.3. Gene Expression Classification

One tissue sample per tumor had been obtained from freshly enucleated eyes by fine needle aspiration of the central tumor region. The contents of the needle hub were transferred into one of two RNAse-free cryovials. Using the same needle, extraction buffer from the second cryovial was aspirated and expelled into the first. This was then placed in a specimen bag, immediately frozen to −80 °C and shipped on dry ice for gene expression classification based on 12 discriminating genes (HTR2B (Gene ID 3357, OMIM 601122), ECM1 (Gene ID 1893, OMIM 602201), RAB31 (Gene ID 1103, OMIM 605694), CDH1 (Gene ID 999, OMIM 192090), FXR1 (Gene ID 8087, OMIM 600819), LTA4H (Gene ID 4048, OMIM 151570), EIF1B (Gene ID 10289), ID2 (Gene ID 3398, OMIM 600386), ROBO1 (Gene ID 6091, OMIM 602430), LMCD1 (Gene ID 29995, OMIM 604859), SATB1 (Gene ID 6304, OMIM 602075), and MTUS1 (Gene ID 57509, OMIM 609589)) and three control genes (MRPS21 (Gene ID 54460, OMIM 611984), RBM23 (Gene ID 55147), and SAP130 (Gene ID 79595, OMIM 609697)). The gene expression classification was performed at a commercial laboratory (Castle Biosciences Inc. Friendswood, TX, USA) which reports three classes of uveal melanoma: Class 2 is associated with a high risk for metastasis, class 1a with a low risk, and class 1b with an intermediate risk [9,20]. Class 2 tumors generally have high relative expression of HTR2B, ECM1, RAB31 and CDH1, whereas class 1 tumors have relatively low expression of these genes and relative high expression of the remaining discriminating genes [20]. All samples had been processed during routine clinical testing for risk prognostication after obtaining patient consent.

### 2.4. Digital Image Analysis

After sectioning and staining, all glass slides were digitally scanned to the ndpi file format at ×400, using identical digital scanners at both institutions (Nano Zoomer 2.0 HT, Hamamatsu Photonics K.K., Hamamatsu, Japan). The digital image analysis software used was the QuPath Bioimage analysis v. 0.2.3, run on a standard off-the-shelf desk top computer (Apple Inc., Cupertino, CA, USA) [21].

In digital image analysis, one BAP-1 positive cell (red chromogen in nucleus) and one negative cell (hematoxylin but no red chromogen in nucleus) was calibrated in each digitally scanned tissue section to adjust for differences in staining intensities and shades between the slides. The tumors were then outlined by drawing of regions of interest. The minimum nucleus area was set to 30 μm^2^ to avoid scoring tumor-infiltrating lymphocytes. The maximum nucleus area was set to 300 μm^2^ and cell expansion to 6 μm, after which the software was observed to correctly outline the borders of tumor cell nuclei and cytoplasm. The positive cell detection function was run with all other parameters left at default (Table S1), under supervision by a board certified pathologist (GS), blinded to gene expression classes and patient outcomes. The staining intensity level of the red chromogen was not taken into account. Any red staining above background was counted as positive. Regions with necrosis, hemorrhage, inflammation, abundant tumor pigmentation and suboptimal staining results as determined by positive and negative internal and external controls were excluded. Hematoxylin and eosin-stained sections were available to help identify appropriate tumor regions for analysis and avoid scoring of lymphocytes, macrophages and other nontumor cells.

A heatmap was then created for each tumor, in which areas with relatively high and relatively low proportions of BAP-1 positive cells were visualized. Several measurements were performed in each tumor to ensure we found the regions with lowest and highest relative expression according to the criteria described below.

The percentage of BAP-1 positive tumor cells was then scored in four different compartments:

(1) Full section. The proportion of BAP-1 positive tumor cells in the entire tumor cross-section.
(2) Hot spot. The circular region with a diameter of 0.5 mm (corresponding to an area of 0.2 mm^2^ per tumor, or one high-power field in a light microscope at 400×) with the highest proportion of BAP-1 positive tumor cells in any part of the tumor.
(3) Cold spot. The circular region with a diameter of 0.5 mm with the lowest proportion of BAP-1 positive tumor cells in any part of the tumor.
(4) Scleral margin. The circular region with a diameter of 0.5 mm with the lowest proportion of BAP-1 positive tumor cells within 1 mm from the tumor base towards the sclera.

The decision to score these four different tumor compartments was based on a method used in breast cancer [22,23]. Previously published methods for BAP-1 scoring in uveal melanoma most commonly focus on tumor areas with the highest relative expression (the hot spots) [7,10,24]. In theory however, the areas with lowest expression (cold spots) may be more prognostically relevant since loss of BAP-1 expression is associated with higher risk for metastasis.

### 2.5. Statistical Methods

Differences with a *p* < 0.05 were considered significant, all *p* values being two-sided. When evaluated by the Shapiro–Wilk test, the deviation from normal distribution was not statistically significant for any of our continuous variables (*p* > 0.05) and all variances were equal (Levene’s test for equality of variances *p* > 0.05). We therefore used independent samples Student’s *t*-test with equal variances assumed when comparing continuous variables between two groups. When comparing means for continuous data in more than two groups, we used one-way ANOVA with 95% confidence intervals (CI). Intratumor differences were calculated and the sensitivity and specificity of the proportion of BAP-1 positive cells in each measured region for gene expression class and development of metastasis were analyzed with receiver operating characteristics. Regression analysis and curve fitting was used to test if the difference in BAP-1 expression between hot spots and cold spots could was a function of tumor volume. The volume of tumors was estimated assuming a semi ellipsoid shape, where *t* is the apical thickness and *lbd* the largest basal diameter of a tumor [12,16]:

$$\mathrm{Volume}\;\mathrm{of}\;\mathrm{tumor}=\frac{\pi }{6}\times t\times lbd^{2}$$

The proportions of BAP-1 positive cells in each region were correlated with Kaplan–Meier metastasis-free survival and Cox regression hazard ratios for metastasis and gene expression class 2. The relative prognostic value of each region was analyzed with likelihood ratio chi-square change (LRΔχ^2^). Median follow-up was defined as the time in months from enucleation to the last occasion metastasis-free patients were known to be alive. Metastasis-free survival was defined as the proportion of patients without metastases to the total number of remaining patients at any point in time. All statistical analyses were performed using IBM SPSS statistics version 26 (Armonk, NY, USA).

## 3. Results

### 3.1. Descriptive Statistics

Of the 40 included patients, 22 were women and 18 men. Their mean age at diagnosis was 64 years (min 24, max 92). Their tumors had a mean thickness of 8.8 mm (standard deviation, SD, 3.4) and a mean diameter of 15.1 mm (SD 3.7). A majority of tumors (21 of 40, 53%) were of American Joint Committee on Cancer (AJCC) tumor size category (T-category) 3a or 3b, and most patients had stage IIB or IIIA disease (29 of 40, 34%) [25]. No patient had radiologically detectable metastases at diagnosis (Table 1).

**Figure 1:**
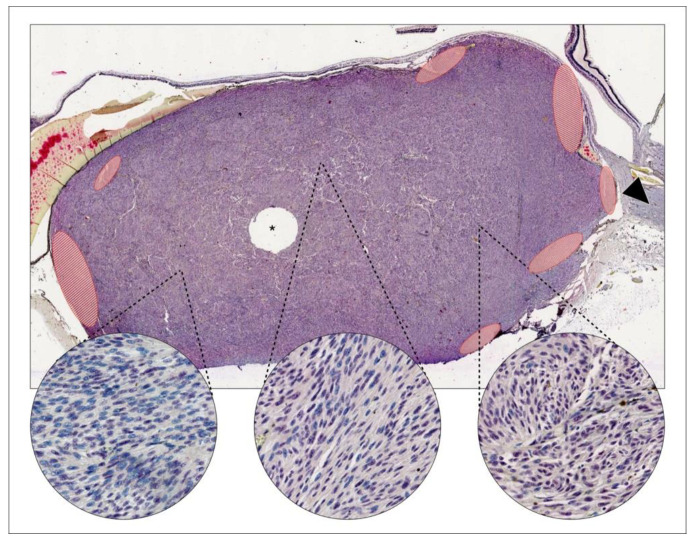
Example of intratumor heterogeneity in BRCA1 associated protein 1 (BAP-1) expression. In a uveal melanoma growing close to the optic nerve head (arrowhead), both BAP-1 positive and negative cells can be identified. Positive nuclei are stained lilac with a red chromogen over blue hematoxylin. Negative cells are stained with blue hematoxylin only. Areas with relatively low (left circle), intermediate (center circle) and high (right circle) proportions of BAP-1 positive cells can be found. Areas with artefacts, uneven staining, necrosis, bleeding, abundant pigmentation or poor focus were excluded from analysis (red-striped areas). Tissue for gene expression classification has been removed from the center of this tumor (asterisk). This case was designated class 1A, implying a low risk for metastasis. Magnification ×20, circles ×400.

**Figure 2:**
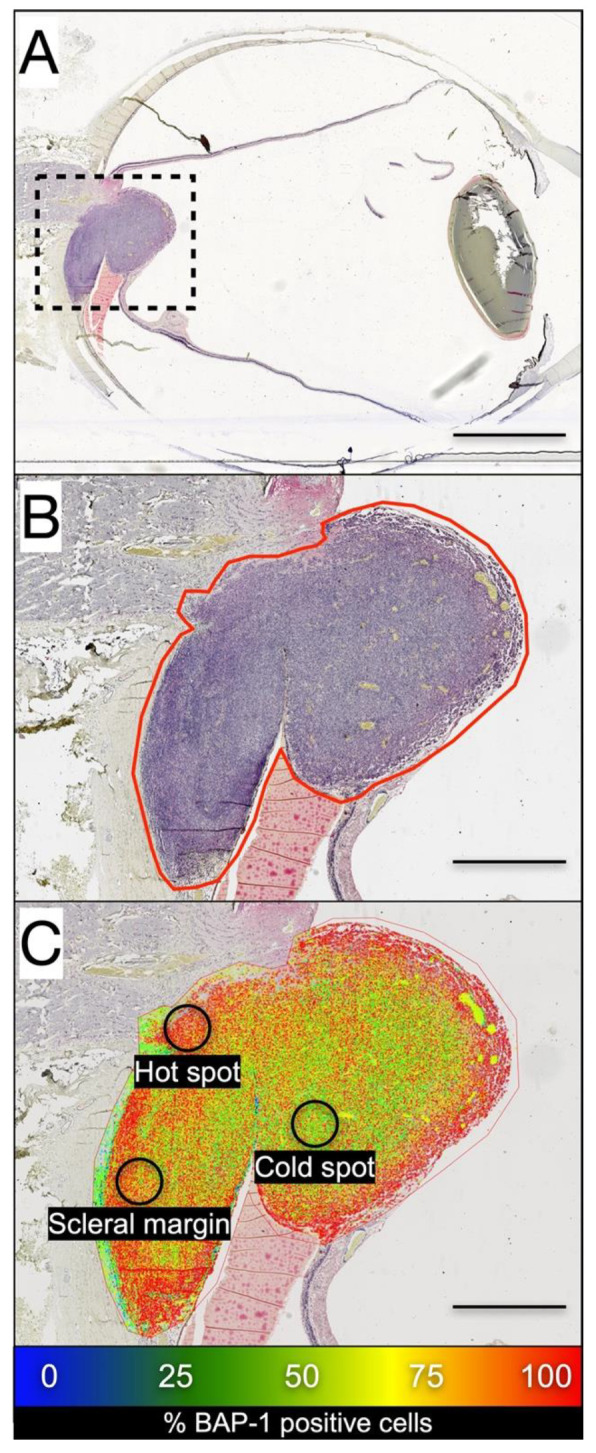
BAP-1 expression heat map by digital image analysis. (**A**) A choroidal melanoma growing on top of the optic nerve head is outlined. (**B**) A region of interest is drawn around the edges of the tumor. In this region, digital image analysis identified BAP-1 positive and negative cells. (**C**) Tumor regions with relatively high (red), intermediate (green) and low (blue) proportions of BAP-1 positive cells can be visualized. The hot spot was defined as the circular region with a diameter of 0.5 mm (corresponding to an area of 0.2 mm^2^ per tumor, or one high-power field in a light microscope at 400×) with the *highest* proportion of BAP-1 positive tumor cells in any part of the tumor. The cold spot was defined as the circular region with a diameter of 0.5 mm with the *lowest* proportion of BAP-1 positive tumor cells in any part of the tumor. Scleral margin was defined as the circular region with a diameter of 0.5 mm with the *lowest* proportion of BAP-1 positive tumor cells within 1 mm from the tumor base towards the sclera. Scale bars: (**A**), 5 mm; (**B**,**C**): 1 mm.

The mean number of cells analyzed in each tumor cross-section was 229 487 (SD 171 818), and in 0.5 mm-diameter hot spots and cold spots 1415 (SD 547). In 22 of 40 tumors (55%), the cold spot was located within 1 mm from the scleral margin (Figure 1 and Figure 2).

Twenty-six patients (65%) were deceased before the end of the study. The median follow-up time for the 14 metastasis-free survivors was 52 months (SD 105).

### 3.2. Intratumor Heterogeneity in BAP-1 Expression

#### 3.2.1. Full Sections

**Figure 3:**
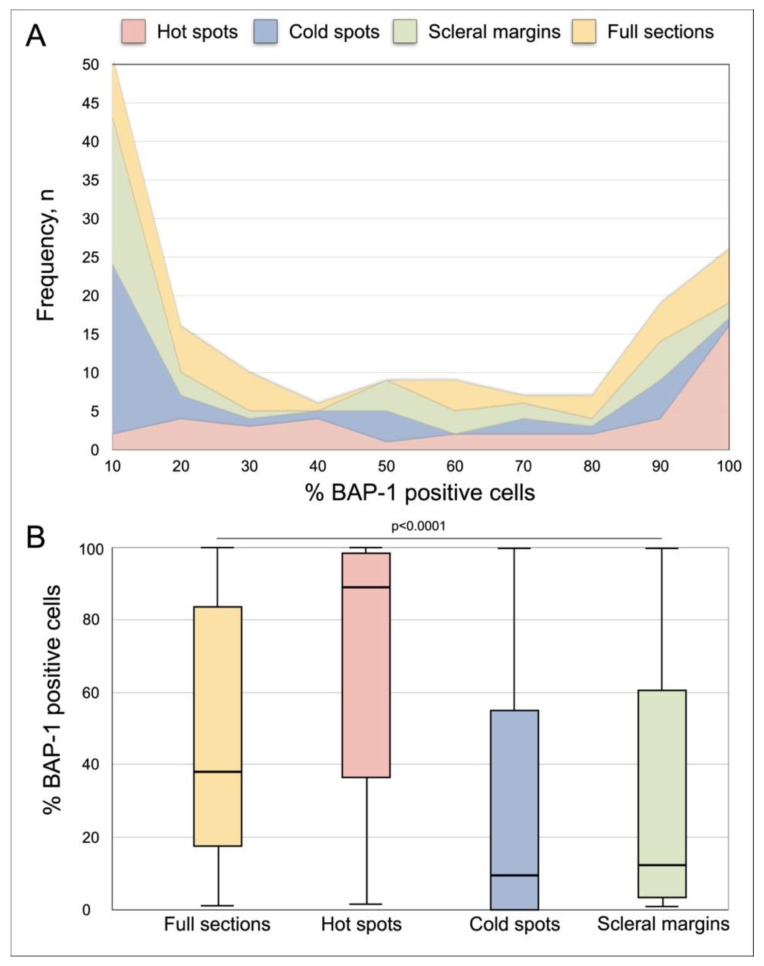
Distribution of the proportion of BAP-1 positive tumor cells in full tumor sections, hot spots, cold spots and scleral margins. (**A**) Cumulative frequency polygon. (**B**) Box plots. The mean proportion of BAP-1 positive cells was significantly different in full sections, hot spots, cold spots and scleral margins (one-way ANOVA *p* < 0.0001). Error bars represent 95% confidence interval.

The mean proportion of BAP-1 positive cells in full tumor sections was 47% (SD 36). In one tumor (3%), 100% of the cells were positive. In eight of 40 tumors (20%), the proportion of BAP-1 positive cells was 90% or more. In another eight tumors (20%), the proportion of BAP-1 positive cells was 10% or less (Figure 3A).

#### 3.2.2. Hot Spots

The mean proportion of BAP-1 positive cells in tumor hot spots was 66% (SD 35). In five of 40 tumors (13%), 100% of the cells were BAP-1 positive. In 17 tumors (43%), the proportion of BAP-1 positive cells was 90% or more. In another two tumors (5%), the proportion of BAP-1 positive cells was 10% or less.

#### 3.2.3. Cold Spots

The mean proportion of BAP-1 positive cells in tumor cold spots was 27% (SD 32). In one of 40 tumors (3%), 100% of the cells were BAP-1 positive with no tumor having 90–99% BAP-1 positive cells. In 22 tumors (55%), the number of BAP-1 positive cells was 10% or less.

#### 3.2.4. Scleral Margins

The mean proportion of BAP-1 positive cells in scleral margins was 32% (SD 34). In one of 40 tumors (3%), 100% of the cells were BAP-1 positive. In two tumors (5%), the proportion of BAP-1 positive cells was 90% or more. In 19 tumors (48%), the number of BAP-1 positive cells was 10% or less.

#### 3.2.5. Heterogeneity

The mean difference in the proportion of BAP-1 positive cells between a tumor’s hot spot and cold spot (intratumor heterogeneity) was 41 percentage points (pp, SD 29).

The mean proportion of BAP-1 positive cells differed significantly in full sections, hot spots, cold spots and scleral margins (one-way ANOVA *p* < 0.0001, Figure 3B).

Tumors with gene expression class 2 had similar intratumor heterogeneity (37 pp difference between hot spots and cold spots, SD 26) as tumors with gene expression class 1a or 1b (39 pp, SD 28, Student’s *t*-test *p* = 0.87). Similarly, intratumor heterogeneity was similar in tumors from patients that later developed metastases (34 pp, SD 26) as in patients that did not (47 pp, SD 31, *p* = 0.19).

In regression analysis, the difference in BAP-1 expression between hot spots and cold spots could was not a function of tumor volume. The relation could not be fitted to linear, logarithmic, inverse, quadratic, cubic, compound, power, S-shaped, growth, exponential or logistic curves (R^2^ < 0.07, F-scores < 1.2, *p* > 0.33).

### 3.3. Sensitivity and Specificity for Gene Expression Class and Metastasis

We analyzed receiver operating characteristics (ROC) of the proportion of BAP-1 negative cells in full tumor sections, hot spots, cold spots and scleral margins with equal emphasis on sensitivity and specificity for the development of metastases and for gene expression class 2.

**Figure 4:**
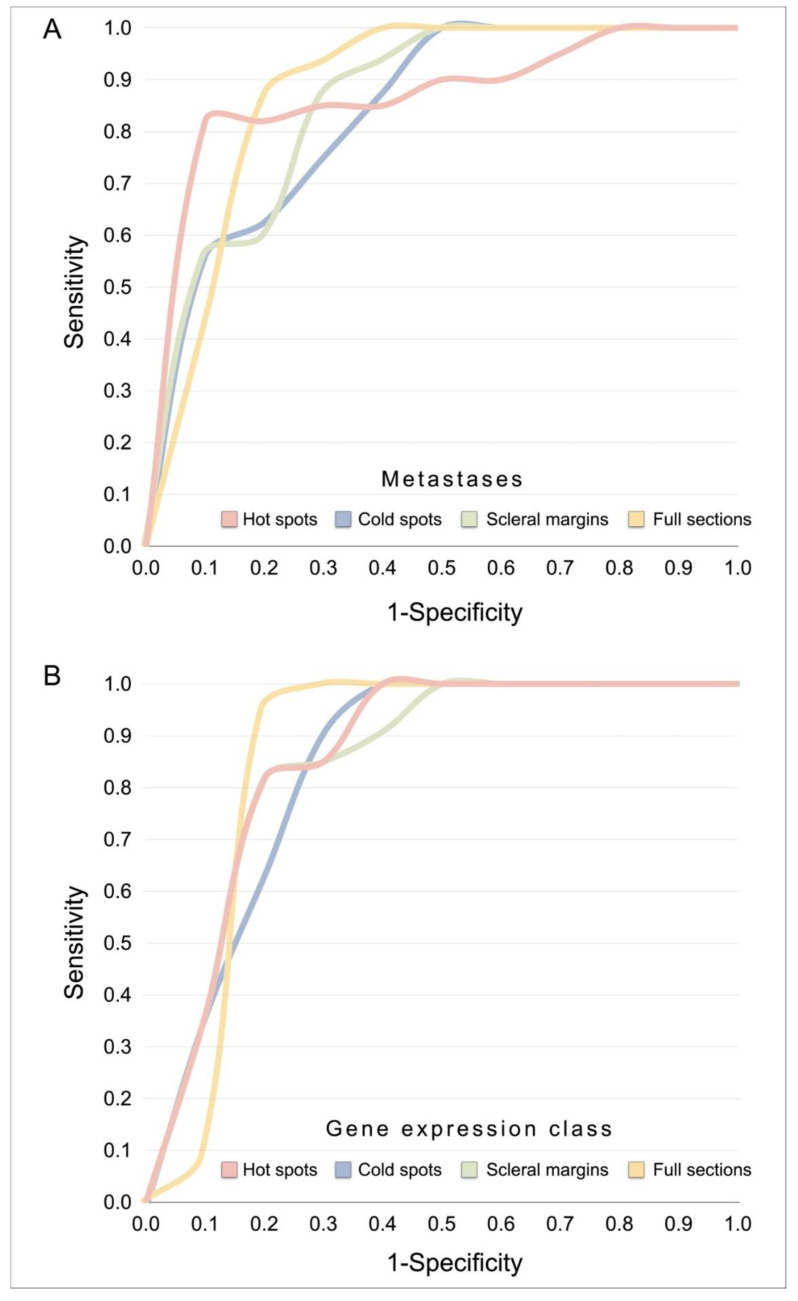
Receiver operating curves. (**A**) For metastases, AUCs of 0.86 to 0.89 were achieved, depending on tumor region (*p* < 0.0001), with sensitivities in the range of 75 to 88% and specificities in the range of 75 to 92%. (**B**) For gene expression class 2, AUCs of 0.85 to 0.86 were achieved (*p* = 0.001 to 0.002), with sensitivities in the range of 82 to 100% and specificities in the range of 73 to 83%. See also Table 2. Abbreviations: AUC, area under the curve.

The area under the curve (AUC), sensitivity and specificity were similar regardless of which tumor region was analyzed, or if metastasis or gene expression class was used as state variable. For metastases, AUCs of 0.86 to 0.89 were achieved (*p* < 0.0001), with sensitivities in the range of 75 to 88% and specificities in the range of 75 to 92% (Table 2a, Figure 4A). For gene expression class 2, AUCs of 0.85 to 0.86 were achieved (*p* = 0.001 to 0.002), with sensitivities in the range of 82 to 100% and specificities in the range of 73 to 83% (Table 2b, Figure 4B).

### 3.4. Survival

**Figure 5:**
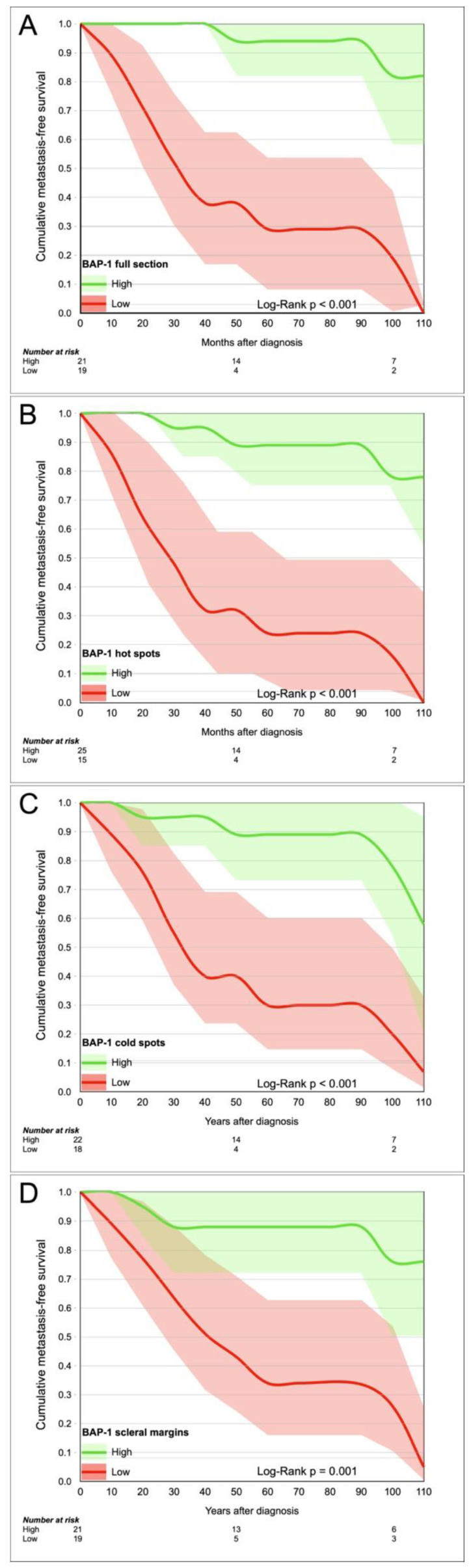
Kaplan–Meier curves. Cumulative metastasis-free survival for patients with high versus low BAP-1 expression, measured in (**A**) full tumor sections, (**B**) hot spots, (**C**) cold spots, and (**D**) scleral margins.

Using the cutoffs for metastasis found in analysis of receiver operating characteristics (Table 2a), we divided tumors into a BAP-1 “high” and “low” group based on the scores in full sections (cutoff 68% negative cells), hot spots (cutoff 46%), cold spots (cutoff 93%) and scleral margins (cutoff 89%). Patients in low BAP-1 groups had significantly shorter Kaplan–Meier metastasis-free survival, regardless of tumor region (Log-Rank *p* ≤ 0.001, Figure 5).

In univariate Cox regression, full sections generated the highest hazard ratios (HR) for metastasis (HR 15.7, *p* < 0.001, 95% CI 3.5 to 70.8). All the other region generated significant but slightly lower HRs (Table 3).

Lastly, we compared the prognostic value of different tumor regions and of tumor size by analysis of likelihood ratio chi-square change (LRΔχ^2^). The prognostic information did not increase when adding BAP-1 expression in full sections to BAP-1 expression in hot spots (LRΔχ^2^ 1.6, *p* = 0.21), but it did increase when adding BAP-1 expression in full sections to cold spots (LRΔχ^2^ 9.5, *p* = 0.002) and to scleral margins (LRΔχ^2^ 9.4, *p* = 0.002).

BAP-1 expression in any tumor region added significant prognostic information to AJCC tumor size category (LRΔχ^2^ 11.6 to 21.0, *p* ≤ 0.001, Table 4a). Similarly, BAP-1 expression in any tumor region added significant prognostic information to gene expression class (LRΔχ^2^ 4.1 to 12.0, *p* < 0.001 to 0.04, Table 4b).

## 4. Discussion

In this paper, the intratumor heterogeneity in uveal melanoma BAP-1 expression is investigated for the first time. Other novelties include a comparison of the relative prognostic significance of measuring this expression in different tumor compartments and of its strong relative prognostic value to tumor size and gene expression classification. We have shown that there is substantial intratumor heterogeneity in uveal melanoma BAP-1 expression. This result is of importance to both ocular surgeons that biopsy uveal melanoma, and to the pathologists that analyze them. Further, even if the eye has been enucleated and the full tumor cross-section is available for immunohistochemical analysis, a pathologist without the aid of digital image analysis cannot realistically evaluate every single cell. Instead, previously published methods for counting the number of BAP-1 positive cells have focused on a smaller fraction of the tumor, e.g., three high power fields or similar [7,10,11,12]. The selection of area for analysis could therefore impact the results of BAP-1 scoring significantly.

However, it should be emphasized that if the threshold for classification is adjusted according to the region analyzed, e.g., a higher threshold for classifying a tumor as BAP-1 positive (with a low risk for metastasis) in hot spots, the intratumor heterogeneity is of no prognostic significance.

Our results confirm the remarkable prognostic value in analysis of BAP-1 expression in uveal melanoma [6,7,8,10,11,12,26]. In fact, it added significant prognostic value to both tumor size category and gene expression class regardless of in which region it was scored.

In a previous publication, we showed that intratumor regions with low BAP-1 expression were more likely to harbor vasculogenic mimicry, had greater microvascular density and a greater number of tumor-infiltrating macrophages [24]. This highlights another aspect of intratumor heterogeneity in uveal melanoma BAP-1 expression: even if loss of BAP-1 expression is a very strong prognostic factor per se, mutant tumor still need a means of intravasating and exiting the eye. We hypothesize that vasculogenic mimicry in tumor regions with high proportions of BAP-1 mutant cells increases metastatic spread by providing a tumor architecture that promotes dissemination [27,28,29].

This study has several limitations. The software used for digital image analysis has cannot distinguish between tumor and nontumor cells including lymphocytes. We tried to limit the impact of this by excluding tumor regions with necrosis, hemorrhage and inflammation, by using hematoxylin and eosin-stained sections to help us select appropriate areas for analysis and by setting the minimum nucleus area to 30 μm^2^ [30]. We also noticed that the software sometimes confused abundant melanin pigment for immunostaining even though a red chromogen was used, resulting in false positives. In these cases, we redefined the positive and negative cell and re-ran the analysis two or three times until the software was observed to identify positive and negative cells correctly. The relatively high proportion of excluded tumors should be noted, as it may have influenced the results. The major reason for the exclusions was the unusually high demands for optimal staining of the entire tumor section. Nevertheless, evaluation of BAP-1 expression would likely benefit from improved and preferably international standardization of BAP-1 immunohistochemistry and tissue fixation protocols. Last, the number of tumors analyzed is relatively limited and it is possible that inclusion of a larger cohort would have influenced the results.

## 5. Conclusions

In conclusion, there is substantial intratumor heterogeneity in uveal melanoma BAP-1 expression. However, this heterogeneity has no prognostic relevance as long as the threshold for classification is appropriately adjusted according to which region is analyzed. Regardless of region, analysis of BAP-1 expression adds significant prognostic value to both tumor size category and gene expression class, adding to the notion that uveal melanoma BAP-1 immunohistochemistry is one of the very strongest prognostic tests in any malignancy.

## Figures and Tables

**Table 1:** Demographics and clinical features of study patients and tumors.

*n* =	40
Mean age at diagnosis, years (min—max)	64 (24–92)
Sex, *n* (%)	
Female	22 (55)
Male	18 (45)
Cell type, *n* (%)	
Spindle	6 (15)
Epithelioid	8 (20)
Mixed	26 (65)
Mean tumor thickness, mm (SD)	8.8 (3.4)
Mean tumor diameter, mm (SD)	15.1 (3.7)
Tumor location, *n* (%)	
Choroid only	31 (78)
Ciliary body only	0 (0)
Iris only	0 (0)
Choroid and ciliary body	7 (18)
Ciliary body and iris	0 (0)
Choroid, ciliary body and iris	2 (5)
Previous brachytherapy, *n* (%)	
No	38 (95)
Yes	2 (5)
AJCC T-category, *n* (%)	
1a–d	1 (3)
2a	8 (20)
2b	2 (5)
2c,d	0 (0)
3a	16 (40)
3b	5 (13)
3c,d	0 (0)
4a	6 (15)
4b	2 (5)
4c,d	0 (0)
AJCC stage, *n* (%)	
I	1 (3)
IIA	8 (20)
IIB	18 (45)
IIIA	11 (28)
IIIB	2 (5)
IIIC	0 (0)
IV	0 (0)
Gene expression class, *n* (%)	
1a	10 (25)
1b	8 (20)
2	9 (23)
Na	13 (33)
Follow-up months, median (SD)	52 (105)

**Table 2:** (**a**). Receiver operating characteristics for the number of BAP-1 negative cells in relation to metastasis of uveal melanoma. * Optimal cutoff for proportion of BAP-1 negative tumor cells in given area. S.E., standard error. C.I., confidence interval. (**b**). Receiver operating characteristics for the number of BAP-1 negative cells in relation to gene expression class 2. * Optimal cutoff for proportion of BAP-1 negative tumor cells in given area. S.E., standard error. C.I., confidence interval.

Tumor Area	AUC	S.E.	*p*	Asymptotic 95% CI		Cutoff *	Sensitivity	Specificity
				Lower Bound	Upper Bound			
(**a**)								
Full section	0.89	0.050	<0.0001	0.80	0.99	68%	88%	79%
Hot spots	0.86	0.065	<0.0001	0.73	0.98	46%	81%	92%
Cold spots	0.86	0.060	<0.0001	0.73	0.96	93%	75%	75%
Scleral margins	0.86	0.058	<0.0001	0.74	0.97	89%	81%	75%
(**b**)								
Full section	0.86	0.078	0.002	0.70	1.00	61%	100%	83%
Hot spots	0.85	0.071	0.002	0.72	0.99	33%	82%	83%
Cold spots	0.85	0.071	0.001	0.71	0.99	93%	82%	78%
Scleral margins	0.86	0.069	0.002	0.73	0.99	91%	82%	73%

**Table 3:** Univariate Cox regressions, hazard for metastasis per BAP-1 status high or low in each tumor region. S.E., standard error. C.I., confidence interval.

Tumor Area	B	S.E.	Wald	*p*	Exp(B)	95% CI	
						Lower	Upper
Full section	2.8	0.8	12.9	<0.001	15.7	3.5	70.8
Hot spots	2.5	0.6	15.1	<0.001	12.4	3.5	43.9
Cold spots	1.8	0.6	9.7	0.002	6.2	2.0	19.5
Scleral margins	1.9	0.6	8.9	0.003	6.8	1.9	24.1

**Table 4:** (**a**). Likelihood ratio chi-square change (LRΔχ^2^) for metastasis when adding BAP-1 expression to American Joint Committee on Cancer (AJCC) tumor size category. (**b**). Likelihood ratio chi-square change (LRΔχ^2^) for metastasis when adding BAP-1 expression to gene expression class.

Tumor Area	LRΔχ^2^	*p*
(**a**)		
Full section	21.0	<0.001
Hot spots	20.9	<0.001
Cold spots	11.6	0.001
Scleral margins	11.8	0.001
(**b**)		
Full section	8.4	0.004
Hot spots	12.0	<0.001
Cold spots	4.1	0.04
Scleral margins	12.0	<0.001

## Data Availability

The data presented in this study are available on request from the corresponding author.

## Supplementary Materials

The following are available online at https://www.mdpi.com/2072-6694/13/5/1143/s1, Table S1: Setup parameters for digital image analysis of uveal melanoma BAP-1 expression.
