# Supplementary material for: Intratumor Heterogeneity in Uveal Melanoma BAP-1 Expression
Source: Cancers (Basel). 2021 Mar 7;13(5):1143. doi: 10.3390/cancers13051143 (PMC7962103; doi:10.3390/cancers13051143)
Supplement: Supplementary file 1 [file cancers-13-01143-s001.pdf]

**Setup parameters for digital image analysis of uveal melanoma BAP-1 expression**	
Requested pixel size	0,5 μm
**Nucleus parameters**	
Background radius	8 μm
Median filter radius	0 μm
Sigma	1,5 μm
Minimum area	20 μm^2
Maximum area	300 μm^2
**Intensity parameters**	
Threshold	0.1
Max background intensity	2
Split by shape	Yes
Exclude DAB	No
**Cell parameters**	
Cell expansion	6 μm
Include cell nucleus	Yes
**General parameters**	
Smooth boundaries	Yes
Make measurements	Yes
**Intensity threshold parameters**	
Score compartment	Nucleus: DAB OD Mean
Threshold 1+	0.2
Threshold 2+	0.4
Threshold 3+	0.6
Single threshold	Yes
